# Cytokine-dependent and–independent gene expression changes and cell cycle block revealed in *Trypanosoma cruzi*-infected host cells by comparative mRNA profiling

**DOI:** 10.1186/1471-2164-10-252

**Published:** 2009-05-29

**Authors:** Jaime A Costales, Johanna P Daily, Barbara A Burleigh

**Affiliations:** 1 Department of Immunology and Infectious Diseases, Harvard School of Public Health, 665 Huntington Ave, Boston, MA, 02115, USA

## Abstract

**Background:**

The requirements for growth and survival of the intracellular pathogen *Trypanosoma cruzi *within mammalian host cells are poorly understood. Transcriptional profiling of the host cell response to infection serves as a rapid read-out for perturbation of host physiology that, in part, reflects adaptation to the infective process. Using Affymetrix oligonucleotide array analysis we identified common and disparate host cell responses triggered by *T. cruzi *infection of phenotypically diverse human cell types.

**Results:**

We report significant changes in transcript abundance in *T. cruzi*-infected fibroblasts, endothelial cells and smooth muscle cells (2852, 2155 and 531 genes respectively; fold-change ≥ 2, p-value < 0.01) 24 hours post-invasion. A prominent type I interferon response was observed in each cell type, reflecting a secondary response to secreted cytokine in infected cultures. To identify a core cytokine-independent response in *T. cruzi*-infected fibroblasts and endothelial cells transwell plates were used to distinguish cytokine-dependent and -independent gene expression profiles. This approach revealed the induction of metabolic and signaling pathways involved in cell proliferation, amino acid catabolism and response to wounding as common themes in *T. cruzi*-infected cells. In addition, the downregulation of genes involved in mitotic cell cycle and cell division predicted that *T. cruzi *infection may impede host cell cycle progression. The observation of impaired cytokinesis in *T. cruzi*-infected cells, following nuclear replication, confirmed this prediction.

**Conclusion:**

Metabolic pathways and cellular processes were identified as significantly altered at the transcriptional level in response to *T. cruzi *infection in a cytokine-independent manner. Several of these alterations are supported by previous studies of *T. cruzi *metabolic requirements or effects on the host. However, our methods also revealed a *T. cruzi*-dependent block in the host cell cycle, at the level of cytokinesis, previously unrecognized for this pathogen-host cell interaction.

## Background

Establishment of infection by intracellular pathogens involves appropriation of host cell functions to facilitate host cell entry, trafficking to specific intracellular locations and for nutrient acquisition [1-5]. Changes in host cell gene expression that accompany the infection process are highly dynamic and reflect a wide array of responses to a specific host-pathogen relationship [6]. As such, transcriptional profiling is frequently used as a genome-wide tool to screen for the impact of pathogens on host cell functions [7-11]. However, deciphering the biological information contained within transcriptional response data is a major challenge in host-pathogen studies [12]. Transcriptional response data provide valuable insights into pathogen-triggered host defense pathways and changes in expression of host cell metabolic genes during infection, the transcriptional outcomes of such events are often obscured by compounded responses to diffusible molecules released by infected or neighboring cells. The ability to identify gene expression changes that occur independently of cytokines and other diffusible molecules provides a first step toward revealing transcriptional changes that report the more intimate host-pathogen interaction, and to providing novel insights into physical and biochemical impact of infection. In this study, we have carried out a comparative analysis of host cell transcriptional response to the intracellular protozoan parasite, *Trypanosoma cruzi*, in three phenotypically diverse mammalian cell types with a view to uncovering a common signature response to parasite infection. We coupled this approach with the use of a transwell plating system to permit the identification of cytokine-dependent and -independent responses to this pathogen.

*T. cruzi*, is the causative agent of human Chagas' disease that affects several million people in South and Central America [13]. Clinical manifestations arise during chronic Chagas' disease and are the result of damage of the cardiac, digestive and/or nervous systems [14]. While the basis for the diverse organ disease patterns has not been elucidated, differential cellular susceptibility to infection, coupled with the nature of the host response elicited in infected cells are likely to influence disease outcome. Trypomastigotes, the tissue invasive forms of *T. cruzi*, are capable of establishing infection within in a variety of nucleated cell types, including professional phagocytes [15] and non-phagocytic cells, such as cardiomyocytes, striated and smooth muscle cells, endothelial cells, adipocytes and neurons [16-19]. To promote entry into non-phagocytic cell types, *T. cruzi *trypomastigotes activate of a number of host cell signaling pathways, including calcium-dependent signaling [20-22], adenylyl cyclase [23], phosphatidylinositol 3-kinases [24,25] and protein tyrosine kinases [26,27]. These early signaling events are thought to orchestrate the host cellular events required for invasion, such as actin microfilament remodeling [28] as well as plasma membrane invagination [29] and targeted lysosome fusion [30,31] that are involved in the formation of the parasitophorous vacuole. *T. cruzi *escapes the vacuole several hours after entry resulting in cytosolic localization of the parasite where transformation to the replicative amastigote stage is completed by 24 hours [32,33]. Over the course of 3–5 days cytosolic amastigotes divide every 12 hours giving rise to several dozen intracellular parasites.

Intracellular growth and persistence of *T. cruzi *amastigotes within a range of host cell types *in vivo *represents an important target for therapeutic intervention, however, little is known regarding the requirements for intracellular growth of *T. cruzi *in mammalian cells. Similar to other kinetoplastid protozoan parasites, *T. cruzi *is a purine auxotroph [34] that can utilize both glucose and amino acids as a carbon source [35]. The inability of *T. cruzi *to synthesize leucine, isoleucine and valine [36] predicts that these aliphatic amino acids are also scavenged from the host. To investigate the impact of intracellular *T. cruzi *infection on host cell gene expression, we compared the global transcriptional response elicited by infection in three different cell types. We find that approximately one-third of the transcriptional changes observed in *T. cruzi*-infected cells at 24 hours post infection, were initiated by cytokines and other diffusible molecules released by infected tissue cultures. The core cytokine-independent response elicited in fibroblasts and endothelial cells underscore metabolic and signaling pathways involved in cell proliferation, amino acid catabolism and response to wounding. Furthermore, the overall dampening of host cell genes related to the mitotic cell cycle and cell division, suggested that *T. cruzi *infection impedes cell cycle progression in the host cell. This prediction was confirmed with the observation of parasite-containing multinucleate cells arising in *T. cruzi*-infected cultures, indicative of a failure to undergo cytokinesis following nuclear replication. Overall, our findings validate the use of transcriptional profiling in conjunction with transwell plates to provide novel insights into biological processes that are modulated in infected host cells in a cytokine-independent manner.

## Results

### *T. cruzi *elicits a robust cytokine-dependent response in diverse cell types

**Figure 1:**
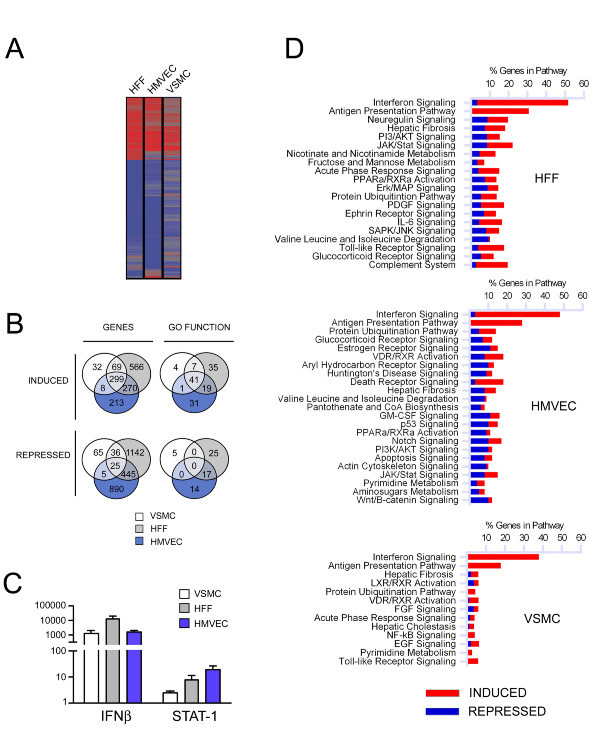
**Differential gene expression in *T. cruzi*-infected HFF, HMVEC and VSMC at 24 hours post-infection**. **A**. Heat map of genes modulated during *T. cruzi *infection in each cell line identified by hybridization to HG_U133 2.0 arrays and analyzed with Rosetta Resolver. Genes modulated (≥ 2-fold, *p *< 0.01) in at least one of the three cell types are shown. **B**. Venn diagrams comparing up or downregulated genes (≥ 2-fold, *p *< 0.01) and GO functions (*p *< 0.05) in each cell type. **C**. qPCR analysis of the expression of interferon beta (IFN-β) mRNA normalized to GAPDH. **D**. Canonical pathways identified by Inguenuity Pathway Analysis™ software as significantly altered (p < 0.05) following *T. cruzi *infection of HFF, HMVEC and VSMC.

To reveal a signature response elicited by *Trypanosoma cruzi *in phenotypically diverse non-phagocytic mammalian cell types, RNA was prepared from mock- and parasite-infected human vascular smooth muscle cells (VSMC), human microvascular endothelial cells (HMVEC) and human foreskin fibroblasts (HFF) at 24 hours post-infection and prepared for hybridization to HG_U133 plus 2.0 Affymetrix arrays for analysis of over 47,000 transcripts. VSMC were found to be less responsive to *T. cruzi *infection than HFF or HMVEC where the average number of gene expression changes observed in two independent experiments was as follows: VSMC: 531 genes (408 up, 131 down); HFF: 2852 genes (1204 up, 1648 down) and HMVEC 2155 genes (790 up, 1365 down) (Figure 1A; see Additional file 1: Table S1, Additional file 2: Table S2, and Additional file 3: Table S3) where differences in the level of infection achieved in each cell type (VSMC: ~75% infected; HFF: ~70% and HMVEC: ~55%) did not obviously correlate with the range or intensity of the transcriptional response in each cell type. No gross variation between the trypomastigote to amastigote transition rate that could account for these differences among cell lines was evident either. The intersection of gene sets representing induced genes in the three cell types revealed 299 genes (Figure 1B; *genes*; see Additional file 4: Table S4). Furthermore, intersecting the GO functions induced in the three cell types, revealed 41 common GO function categories (Figure 1B; *GO functions; *see Additional file 5: Table S5). Within this common set of genes, interferon-stimulated genes (ISGs) including IFN-stimulated exonuclease (ISG20), 2,5-oligoadenylate synthase (OAS1) and myxovirus resistance protein (MX1), featured as among the most highly expressed in parasite-infected VSMC, HFF, and HMVEC (see Additional file 1: Table S1, Additional file 2: Table S2 and Additional file 3: Table S3, respectively). As a reflection of the robust induction of ISGs, 'IFN-signaling' was identified as the top signaling pathway induced by *T. cruzi *in infected cells (Figure 1C) consistent with the marked increase in IFNβ expression in all cell types (Figure 1D).

### Dissecting the global host cell transcriptional response to *T. cruzi*

**Figure 2:**
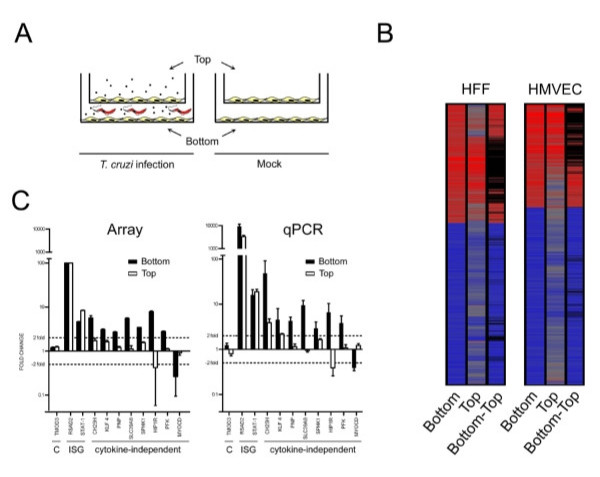
**Identification of cytokine-dependent and – independent transcriptional responses in *T. cruzi*-infected HFF and HMVEC**. **A**. Schematic showing experimental design where *T. cruzi*-infected cells on the bottom of the transwell plate are separated from uninfected cells on the top layer and mock-infected controls. **B**. Heat maps showing combined information for two replicate experiments for changes in transcript abundance (≥ 2-fold, p < 0.01) for cells plated in the bottom and top the top Transwell insert and for the genes remaining after subtraction of the genes present in both top and bottom (Methods). Black bars represent cytokine-dependent genes removed by filtering the top response from the bottom. **C**. qPCR confirmation of microarray data for selected non-differentially expressed genes (*C*), IFN-stimulated genes (*ISG*) and presumptive cytokine-independent genes (*cytokine-independent*) identified by microarray analysis of samples obtained from HFF cells in Transwell experiments. Where more than one sequence corresponding to the same gene was modulated in the microarray data, the perfect match (_at) sequence was chosen. Tropomodulin 3 (TMOD 3), IFNβ (IFN-β), radical S-adenosyl methionine domain-containing protein 2 (RSAD2), signal transducer and activator of transcription 1 (STAT-1), cholesterol-25-hydroxylase (CH25H), kruppel-like transcription factor-4 (KLF4), purine nucleoside phosphorylase (PNP), solute carrier family 39 member 8 (SLC39A8), sphingosine kinase-1 (SPHK1), huntingtin-interacting protein 1-related protein (HIP1R), phosphofructokinase (PFK), myocardin (MYOCD). cDNA generated from three independent infections was analyzed and the mean fold-change ± s.d. are reported for the qPCR.

While the induction of cytokines and the cytokine-dependent response in infected host cells provides important information regarding the nature of the innate immune response to *T. cruzi*, gene expression changes occurring in a cytokine-independent manner are predicted to more directly report the impact of intracellular parasitism. In addition to IFNβ, *T. cruzi *triggers the expression of cytokines such as IL-6, IL-8, IL-11 and IL-15 in host cells (see Additional file 6: Table S6), which are predicted to contribute to the global transcriptional response to this pathogen. To discriminate between cytokine-dependent and cytokine-independent responses to *T. cruzi*, a transwell plating system was adopted to experimentally dissect the global transcriptional response in HFF and HMVEC. In this setup, cells in the top chamber serve as reporters for the effect of cytokines and other diffusible factors (herein referred to as 'cytokines' for simplicity) secreted by parasite-infected cells in the bottom chamber (Figure 2A). (VSMC were omitted from the transwell experiments due to difficulties obtaining adequate RNA from cells plated on the top chamber.) As predicted, cells exposed only to secreted cytokines and diffusible products (Figure 2A, B, *Top*) generated by the infected cell population (Figure 2A, B, *Bottom*) responded with significant changes in gene expression. The cytokine-responsive genes represent a substantial fraction of the total number of genes modulated in infected cells: 28% in HFF (803 of 2852 genes) and 22% in HMVEC (488 of 2155 genes).

**Figure 3:**
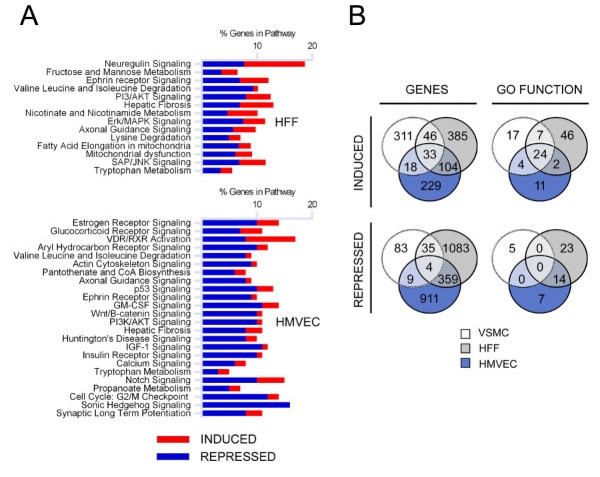
**Cellular processes predicted by microarray data to be modulated during *T. cruzi *infection**. A. Canonical pathways in *T. cruzi*-infected cells that are significantly modulated in a cytokine-independent manner. B. Venn diagrams comparing genes and representative GO functions for all up or downregulated genes (p < 0.05) in VSMC or following subtraction of the cytokine-dependent genes identified in the transwell experiments for HFF and HMVEC.

To arrive at a subset of cytokine-independent gene changes, the cytokine-responsive genes (Figure 2B, *Top*) were filtered from the set of genes changing ≥ 2-fold in parasite-infected cells (Figure 2B, *Bottom*) as displayed (Figure 2B, *Bottom-Top*; (see Additional files 7: Tables S7 and Additional files 8: Table S8 for gene lists). The resultant heat map clearly shows that a large number of genes, including those annotated as IFN-responsive, were removed by this filtering step (see Additional files 7: Tables S7 and Additional files 8: Table S8). In addition, the biological pathways corresponding to 'IFN signaling', 'antigen presentation' and 'protein ubiquitination' that featured prominently in the overall transcriptional response to *T. cruzi *(Figure 1C), are no longer represented as part of a significant cytokine-independent response in HFF and HMVEC (Figure 3A). Quantitative RT-PCR (qPCR) was used to confirm some of the cytokine-dependent and -independent predictions from the microarray analysis with excellent concordance (Figure 2C). Of note, many of the cytokine and chemokine genes induced upon infection (eg. IFNβ, IL-8, IL-11, IL-15 and CCL5) were found to be cytokine-independent (see Additional file 6: Table S6), indicating that the expression of some cytokines and chemokines likely occurs as part of the primary response to *T. cruzi*. Others, such as IL-6, appear to be upregulated in response to diffusible molecules in parasite-infected cultures.

### *T. cruzi *alters expression of host cell metabolic and signaling pathways in a cytokine-independent manner

To uncover a cytokine-independent signature response to *T. cruzi *that is shared by diverse host cell types, we identified metabolic and signaling pathways that are significantly modulated in response to infection after filtering out the cytokine-dependent genes (Figure 3A). In general, little overlap was observed in host cell pathways found to be significantly perturbed in *T. cruzi*-infected HFF and HMVEC (Figure 3A). However, pathways involving "valine, leucine and isoleucine degradation", "ephrin receptor signaling" and "PI3/AKT signaling" were similarly altered in both *T. cruzi*-infected HFF and HMVEC (Figure 3A). Several enzymes in the valine, leucine and isoleucine degradation pathway were downregulated in both cell types including BCAT2, which catalyzes the first step in the degradation of branched chain amino acids [37]. In the PI-3 kinase signaling pathway, two negative regulators of Akt, PP2A and CTMP were downregulated in infected fibroblasts and endothelial cells, as was TSC2, a negative regulator of the mTOR pathway. In the ephrin receptor signaling pathway, upregulation of growth factors such as FGF, PDGF and VEGF that signal through receptor tyrosine kinases and repression of heterotrimeric G proteins in HFF whereas in HMVEC, there is a trend toward downregulation of growth factor signaling pathways and a similar repression of heterotrimeric G proteins.

A set of 500 genes was found to be commonly altered in a cytokine-independent manner in response to *T. cruzi *infection of HFF and HMVEC (Figure 3B; genes) which was represented by 59 GO function categories (Figure 3B; GO functions) (see Additional file 9: Table S9). The top functional categories associated with upregulated genes in HFF and HMVEC included 'immune response' and 'response to wounding' (see Additional file 10: Table S10 for genes). To extend our analysis of shared host cell responses to *T. cruzi*, we next considered transcriptional response data for VSMC despite the lack of transwell data for this cell type. Intersection of VSMC expression data with cytokine-independent genesets from HFF and HMVEC reveals only 37 genes (33 induced; 4 repressed) that are commonly modulated in the three cell types following infection with *T. cruzi *(Table 1). In addition to strong expression of IFNβ and CCL5 in all cell types, parasite infection induces the expression of genes involved in cell-matrix interactions (osteopontin; SPP1) carbohydrate modification (B4GT5 and B3GNT2), vesicular transport SYTL3 and T-SNARE1 and Ca2+ homeostasis STIM1 and CARKL (Table 1). In terms of common metabolic genes, pantothenate kinase 2 (PANK2), the first enzyme in the biosynthetic pathway for coenzyme A production, was upregulated in these diverse cell types in response to *T. cruzi*-infection. While these genes do not assemble into common metabolic or signaling pathways, examination of significant GO terms indicate that the most basic common features of the host cell response to *T. cruzi *infection of fibroblasts, endothelial cells and smooth muscle cells involve the induction of stress response genes and those involved in cell growth and proliferation (Table 2).

**Table 1:** Common cytokine-independent gene expression changes in *T. cruzi*-infected cells.

**Name**	**Sequence Description**	**Accession**	**HFF Fold**	**HFF** **p-value**	**HMVEC Fold**	**HMVEC** **p-value**	VSMC Fold*	**VSMC** p-value*
IFNB1	Interferon, beta 1, fibroblast		100	0.00E+00	98.6	4.50E-27	11.4	3.11E-06

CCL5	Chemokine (C-C motif) ligand 5	AF043341	71.0	9.13E-35	57.1	4.53E-19	36	5.38E-19

CCL5	Chemokine (C-C motif) ligand 5	M21121	35.9	4.30E-16	58.2	4.53E-08	36.7	2.23E-15

CCL5	Chemokine (C-C motif) ligand 5		33.5	1.96E-25	92.0	8.48E-16	27.4	1.00E-13

LOC126520	Hypothetical protein	AK054808	27.9	9.07E-18	11.4	8.23E-14	6.7	3.00E-05

215941_at	MRNA, Xq terminal portion	D16471	10.6	8.07E-07	9.1	8.84E-14	9.9	4.30E-08

LOC400740	Hypothetical gene	AW205774	10.4	6.68E-11	5.1	1.08E-03	6	8.50E-04

ZBTB7A	Zinc finger and BTB domain containing 7A	AI568395	8.8	3.20E-15	17.3	8.61E-19	11	6.11E-06

SSH2	Slingshot homolog 2 (Drosophila)	AB072358	8.4	5.85E-10	8.5	8.02E-08	5.6	1.57E-03

CARKL	Homo sapiens carbohydrate kinase-like (CARKL)		8.4	4.72E-08	9.0	4.64E-08	4.5	6.80E-03

1565716_at	GN0053 Homo sapiens cDNA, mRNA sequence.	BE930017	7.9	3.38E-18	10.1	2.29E-14	4.2	3.00E-05

SLC24A4	SLC family 24 (sodium/potassium/calcium exchanger), member 4	W90718	7.4	1.02E-08	4.7	2.95E-06	5.9	5.20E-04

LOC283761	Hypothetical protein	BC039350	6.5	3.30E-04	15.3	2.20E-08	11	4.57E-06

SYTL3	Synaptotagmin-like 3	AI990716	6.5	1.47E-06	4.8	1.00E-05	4.3	4.67E-03

FLJ35390	Hypothetical protein	BC024303	6.4	2.22E-10	6.2	3.83E-07	2.8	3.35E-03

IL17RE	Similar to contains element TAR1 repetitive element	AW003256	6.1	1.45E-06	3.4	2.74E-03	4.7	8.01E-03

SIM2	Single-minded (Drosophila) homolog 2 short isoform		5.8	7.69E-03	7.1	8.47E-06	6.8	1.48E-03

FLJ14213	Hypothetical protein	AV732741	4.8	4.02E-03	6.6	2.50E-04	10.3	1.10E-04

231673_at	Transcribed locus	AW273730	4.7	7.94E-06	5.6	3.00E-05	2.7	7.29E-03

TSNARE1	T-SNARE domain containing 1	AI741779	4.6	7.19E-13	3.5	8.00E-05	3.8	3.00E-05

234510_at	cDNA clone DKFZp566F133	AL049357	4.0	6.70E-04	4.2	9.00E-05	4.7	3.71E-03

PANK2	Pantothenate kinase 2	AV703394	3.5	9.91E-08	3.7	2.78E-03	2.1	7.06E-03

B3GNT2	beta-1,3-N-acetylglucosaminyltransferase		3.4	2.77E-09	2.9	3.56E-03	2.1	3.00E-05

241657_at	Transcribed locus	AI791835	3.3	1.21E-03	4.4	8.50E-04	3.7	8.99E-03

STIM1	Stromal interaction molecule 1	BC016014	3.1	2.30E-04	3.9	1.51E-08	2.6	4.81E-03

KLF4	Kruppel-like factor 4	BF514079	3.1	1.74E-08	8.2	1.04E-03	3.4	2.20E-07

HIST1H2AJ	H2A histone family, member E		3.1	1.22E-09	4.7	1.85E-09	2.1	2.40E-04

HIST1H2AI	H2A histone family, member C		3.0	2.69E-07	4.9	5.39E-08	2.1	4.60E-04

FNDC3A	Cytochrome c oxidase subunit VIIc pseudogene 1	AL137000	2.8	5.61E-08	2.2	2.59E-03	2.3	4.03E-06

B4GALT5	beta 1,4-galactosyltransferase polypeptide 5	AL035683	2.8	4.55E-24	2.0	1.60E-10	2.1	2.38E-08

SPP1	Secreted phosphoprotein 1 (osteopontin)	M83248	2.6	6.17E-03	5.1	1.80E-04	3.1	4.40E-04

CD55	Decay accelerating factor for complement		2.5	2.00E-05	2.2	9.37E-06	2.2	2.05E-06

LYSMD2	LysM, putative peptidoglycan-binding, domain containing 2	AI674731	2.1	3.00E-05	3.5	3.58E-25	2.9	1.00E-05

PTPN11	Protein tyrosine phosphatase, non-receptor type 11	L07527	-2.3	7.47E-03	-5.2	7.40E-04	-2.2	1.04E-03

PKP4	Plakophilin 4		-2.5	4.14E-08	-2.4	4.80E-04	-2	1.60E-07

MGC52110	hypothetical protein MGC52110	BF195431	-4.0	1.01E-06	-4.9	7.67E-06	-2.3	4.33E-03

MYT1	Myelin transcription factor 1	AB028973	-5.3	2.37E-16	-4.4	6.57E-06	-2.1	5.52E-03

Gene expression changes occurring in human vascular smooth muscle cells 24 hours after infection with *T. cruzi*. The data shown corresponds to probes for transcripts showing an intensity increase of 2-fold or greater (p < 0.01) with respect to their matched controls according to the analysis with Rosetta Resolver Biosoftware 7.0. *Transwell data not available for VSMC.

**Table 2:** Cytokine-independent biological functions modulated during *T. cruzi *infection.

	**HFF** **p-value**	**HFF** **# genes**	**HMVEC** **p-value**	**HMVEC** **# genes**	**VSMC** **p-value**	**VSMC** # genes*
**UPREGULATED** **GO CATEGORY**						

GO:9611: response to wounding	9.88E-09	29	9.25E-05	17	2.18E-05	17

GO:6955: immune response	1.02E-06	40	8.60E-05	27	2.01E-32	64

GO:42127: regulation of cell proliferation	1.76E-06	24	4.50E-04	15	4.37E-04	14

GO:6950: response to stress	1.45E-04	44	4.23E-05	35	6.12E-13	48

GO:6915: apoptosis	8.56E-03	24	1.38E-04	23	3.06E-03	18

**DOWNREGULATED** **GO CATEGORY**						

GO:7049: cell cycle	1.08E-05	78	2.03E-05	77	- -	- -

GO:6412: protein biosynthesis	1.51E-02	48	2.57E-02	47	- -	- -

Shown are selected GO functions modulated (p < 0.05, overlap ≥ 5 genes) in a cytokine-independent fashion 24 hours after *T. cruzi *infection.*Transwell data was not available for VSMC.

### *T. cruzi *infection impedes late mitotic events

**Figure 4:**
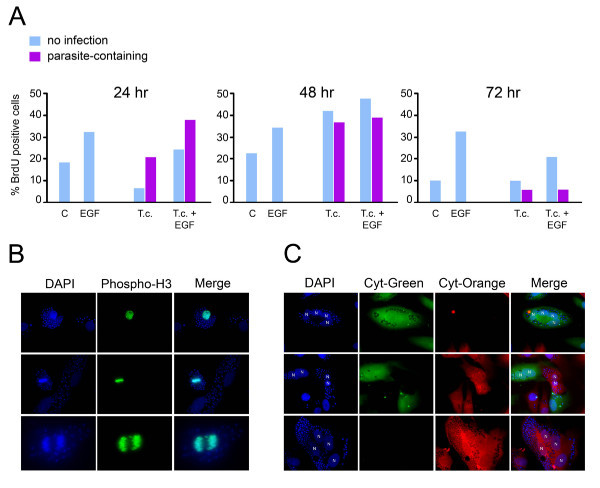
**Cell cycle progression of *T. cruzi*-infected cells**. **A**. Quantitation of BrdU-stained HFF cells mock- or *T. cruzi*-infected in the presence or absence of 5 ng/ml EGF for 24, 48 or 72 hours. Within infected cultures, parasite-containing and parasite-free cells were scored separately. **B**. Phospho-H3 staining (green) in *T. cruzi*-infected cells at 48 hours post-infection where host and parasite DNA are stained with DAPI (blue). **C**. Images of co-cultured *T. cruzi*-infected HFF cells preloaded with Cytotracker™-green (green) or Cytracker™-orange (red). Host cell nuclei (N) and parasite DNA (smaller blue) are stained with DAPI (blue).

GO function analysis of genes that are downregulated in *T. cruzi*-infected HFF and HMVEC in a cytokine-independent manner, reveal a significant bias toward 'mitotic cell cycle', 'M phase', and 'cell division' (see Additional file 9: Table S9), suggesting the possibility that *T. cruzi *infection negatively impacts the host cell cycle. To further characterize this finding, markers of cell cycle progression were evaluated in *T. cruzi*-infected cells. We first examined the relative ability of infected cells to incorporate BrdU into nuclear DNA (marking S-phase) by immunofluorescence microscopy where parasite-containing and parasite-free cells were scored separately. *T. cruzi*-containing cells incorporate BrdU into nuclear DNA at levels similar to mock-infected controls at 24 hours and by 48 hr post-infection, twice as many infected cells were positive for BrdU (~40%) as compared to controls (~20%) (Figure 4A) revealing that parasite infection does not impair cell entry into S-phase. Given that this increase was observed in both uninfected and parasite-containing cells in the monolayer (Figure 4A, 48 hr) suggests the presence of a soluble 'mitogen' in infected cell cultures that stimulates DNA replication in neighboring uninfected cells. In contrast, by 72 hr post-infection nuclear BrdU incorporation was shown to be minimal in parasite-infected cells where increases in incorporation were found to be refractory to EGF stimulation (Figure 4A, 72 hr). However, the uninfected cells in the population were still able to respond to EGF with increased BrdU incorporation relative to parasite-containing cells. These data suggest that following an increase in cell cycle progression, a parasite-specific block on cell cycle progression occurs somewhere between 48 and 72 hr post-infection.

The ability to label host cell nuclei with anti-phospho-H3 at 48 hours post-infection (Figure 4B) indicates that *T. cruzi*-infected cells are not blocked at the G2/M phase. However, in 48 and 72 hour cultures *T. cruzi*-infected cells containing 2, 3 or 4 nuclei were observed (Figure 4C) where the multinucleate phenotype was strictly observed in parasite-containing cells in the infected cultures. No multinucleate cells were observed in mock-infected control populations at any time point (data not shown). Because multinucleate cells can arise as a result of a block in cytokinesis following nuclear division or via cell-to-cell fusion, fluorescently labeled cells were used to distinguish between these possibilities. Cells labeled with Cytotracker-green or Cytotracker-orange were infected in separate dishes for 2 hours then mixed in a 1:1 ratio and infection was allowed to progress for 48 and 72 hours. Parasite-containing multinucleate cells were either green or orange as shown in the representative images (Figure 4C). No yellow cells, indicating that fusion had occurred, were observed under any condition. These data indicate that the multinucleate cells arose from rounds of nuclear duplication without cytokinesis, demonstrating the inhibitory effect of *T. cruzi *infection on the late stages of host cell division as predicted from the results of the microarray analysis.

## Discussion

Perturbation of host metabolic pathways and cellular functions following pathogen infection is predicted to result in compensatory changes in the expression of key regulatory components in at least some of the affected pathways. To facilitate elucidation of these critical functions in *T. cruzi*-infected host cells, we identified a core transcriptional response elicited in phenotypically diverse cell types following intracellular infection with this pathogen. A subset of host cell genes for which expression was similarly modulated in three different human cell types was identified in dermal fibroblasts, microvascular endothelial, and vascular smooth muscle, following infection with *T. cruzi*. Within this subset of genes we find that interferon-stimulated genes (ISGs) are among the most highly induced by *T. cruzi *in the three cell types. This finding is consistent with previous observations that *T. cruzi *triggers IFNβ expression in fibroblasts [38], macrophages and dendritic cells [39] and reveals the robust nature of the cytokine-driven response in shaping the early transcriptional profile to this pathogen. In the second phase of the study, transwell plates were employed to experimentally distinguish cytokine-dependent and -independent responses elicited in *T. cruzi*-infected cells. This application permitted the identification of metabolic pathways and cellular processes that were significantly altered by the infective process in a cytokine-independent manner, enabling us to focus on host cell transcriptional changes that relate more directly to the metabolic impact of intracellular parasitism.

Host transcriptional responses to *T. cruzi *become detectable at 24 hours post infection [38] which reflect the cumulative pre- and post-invasion events including differentiation of the invasive *T. cruzi *trypomastigotes to replicative amastigotes located in the host cell cytoplasm. It is predicted that these activities are responsible for a subset of the cytokine-independent changes in expression of host biosynthetic pathways such as those that would favor utilization of metabolites by the parasite. For example, in parasite-infected fibroblasts, endothelial cells and smooth muscle cells we observe upregulation of host 5'-nucleotidase and/or purine nucleoside phosphorylase (PNP), which generate the purine nucleosides and purine bases [40]. Given that *T. cruzi *is reliant on purine salvage for growth [41], induction of purine catabolic enzymes in infected host cells may directly benefit replicating parasites. Along these lines, where *T. cruzi *is incapable of synthesizing the aliphatic amino acids, leucine, isoleucine and valine [36], we have observed a general repression of the valine, leucine and isoleucine degradation pathway, including expression of the first enzyme in the catabolic pathway, BCAT2. Inhibiting the breakdown of these amino acids would presumably increase their cellular concentration and opportunities for scavenging by the parasite. Another gene of interest, upregulated in diverse cell types in response to *T. cruzi*, is PANK2, that encodes pantothenate kinase 2, the first enzyme in the biosynthetic pathway for Coenzyme A production [42]. Given that CoA is required for GPI anchor synthesis, which is the modification of choice for *T. cruzi *surface glycoproteins, it is tempting to speculate that by co-opting host CoA or one of its precursors, the parasite creates an increased demand for CoA which is met by upregulating this key enzyme in the host cell CoA biosynthetic pathway.

In addition to potential changes in host cell biosynthetic activities, the expression of apoptotic regulators and stimulators of cell proliferation were also significantly induced in *T. cruzi*-infected cells. This observation is interesting in light of data showing that *T. cruzi *infection protects cardiomyocytes and neuronal cells from apoptotic stimuli [43,44]. Moreover, parasite-mediated protection from cell death appears independent of diffusible molecules released from infected host cells [44], consistent with the observed modulation of the expression of apoptosis regulators in a 'cytokine-independent' manner in this study. While the mechanistic basis for the apoptotic block is currently not known, alteration of expression of host cell genes related to apoptosis is likely to benefit the parasite by preventing host cell death before the intracellular replicative cycle is complete.

Despite commonalities observed in the early response of diverse host cell types to *T. cruzi*, a minor fraction of host metabolic and signaling pathways were found to be shared by *T. cruzi*-infected fibroblasts, endothelial and smooth muscle cells. Intrinsic differences in the levels of available host cell metabolic intermediates might be a determining factor in the activation of homeostatic mechanisms and associated transcriptional changes. In addition to absolute differences between cell types, kinetic differences in the cellular response to metabolic pressures exerted by intracellular parasites may also produce non-overlapping responses at a given time-point. In our study, which examined only 24 hours post-infection, VSMC were found to respond with relatively few changes in transcript abundance as compared to HFF or HMVEC, despite similar burden of intracellular parasites. A comparison of canonical pathways altered in VSMC at 48 hr post-infection (for which there were >2000 genes as compared to ~500 observed at 24 hours, see Additional file 11: Table S11) with the cytokine-independent response triggered in HFF and HMVEC revealed an increase in the number of overlapping pathways (not shown). This observation suggests that cell type specific responses to *T. cruzi *include a kinetic component that influences the timing for which significant changes are observed in an infected cell type. In general, presumptive compensatory changes in host cell genes involved in certain metabolic processes: *eg*. purine metabolism, aliphatic amino acid catabolism, cell growth and anti-apoptosis might have been predicted based on our knowledge of *T. cruzi *physiology and interactions of the parasite with the host cell. However, our array analysis also predicted the unexpected finding that *T. cruzi *infection might impede cell cycle progression, on the basis that the top GO terms associated with downregulated cytokine-independent genes in HFF and HMVEC were associated with mitosis and cell division. While the impact of *T. cruzi *on cellular proliferation genes has been noted in a previous microarray study of *T. cruzi*-infected HeLa cells at 72 hours post-infection [45], no conclusions were drawn as to the impact of these changes on cellular proliferation. Here, we have demonstrated that *T. cruzi*-infected fibroblasts are capable of DNA synthesis and nuclear replication, but exhibit abortive cytokinesis as judged by an accumulation of multinucleate cells at 48 and 72 hours post-infection. The increased level of BrdU incorporation observed in both parasite-containing and parasite-free cells in infected monolayers at 48 hours post-infection suggest that infection with *T. cruzi *potentiates host cell DNA synthesis early in the infective process and, secondly, that this may be mediated by a soluble factor in the medium. These findings are consistent with the observation that a number of growth factor genes (FGF, PDGF, VEGF) are upregulated in infected fibroblasts at 24 hours post-infection and that 'cellular proliferation' features as one of the overrepresented GO terms for upregulated genes in *T. cruzi*-infected HFF, HMVEC and VSMC.

The mechanistic basis for the block in cell division is currently unknown, but it may be linked to a general dampening of key cytoskeletal genes that would be required for cytokinesis, including septins and several actin regulatory genes (ARPC1A, DLG1, FARP1, MYH10, WIRE) as well as induction of the protein phosphatase slingshot-2 (SSH2) in infected cells which promotes actin disassembly via the activation of cofilin [46]. Related to these findings, we have recently demonstrated that the mechanical properties of *T. cruzi *infected cells change significantly with infected cells becoming less stiff over the course of a 72 hour infection in a manner that appears to be related to decreased Rho kinase activity in infected cells [47]. While the significance of these findings is presently unclear, it is possible that inhibition of host cell division provides a more favorable environment for nutrient acquisition by the intracellular parasites. It should be noted that some of the important *in vivo *targets of *T. cruzi *infection, cardiomyocytes for example, are terminally differentiated cells that are no longer undergo mitosis. It has also been demonstrated that mitotic cells are relatively refractory to infection *in vitro *[48]. While there are many potential reasons for this observation, including the dramatic reorganization of microtubules and reduced vesicular trafficking that occurs during this stage of the cell cycle [49], the collective set of observations are reflective of the important interplay between host cell cycle and *T. cruzi *infection. Several intracellular parasites are known to impinge on host cellular proliferation pathways where *Leishmania amazonensis *and *Toxoplasma gondii *block host cell cycle progression at the G1/S transition [50] and G2/M stages [51,52], respectively. In contrast, *Theileria parva *promotes uncontrolled proliferation of infected host lymphocytes [53]. The ability of *Toxoplasma *to block cell cycle progression was associated with the induction of host UHRF1 protein (ubiquitin-like, containing PHD and RING finger domains 1), the suppression of which by siRNA blocks both G2 arrest and reduces parasite proliferation in the host cell [52]. A similar induction of UHFR1 mRNA is not observed at 24 hours post-infection at any of the cell types infected with *T. cruzi*, consistent with the observation that this parasite does not impede cycles of DNA replication and nuclear division, but instead, appears to dysregulate aspects of cytokinesis. Additional studies will be instrumental for elucidation of the mechanism of *T. cruzi*-mediated interruption of the host cell cycle and to determine the benefits, if any, to host and pathogen. Such studies could include transcriptional profiling of other cell types, including cardiomyocytes and other non-dividing cells, which constitute important targets for *T. cruzi *infection, and would provide a contrasting view with respect to the cells that were examined here. Furthermore, although the cell cycle progression analysis was performed in cell cultures that had reached confluence 48 hours prior the experiments and demonstrated to be were arrested in G0 (data not shown), future global transcriptional profiling of synchronized cells could more clearly define the modulation of cell cycle-related genes. Prior to this study, there was no indication in the literature that *T. cruzi *can block host cell division. The novel findings, reported here, demonstrate the utility of transcriptional profiling in directing the rational search for host biological functions affected during pathogen intracellular infection.

In summary, we have uncovered novel host cell physiology secondary to parasite infection that is cytokine-independent, including evidence for parasite induced host cell cycle arrest. Given that we have limited knowledge of the intracellular requirements for *T. cruzi *amastigote replication and survival, the parasite-modulated pathways identified here represent potential targets for further functional analysis and intervention.

## Conclusion

**Figure 5:**
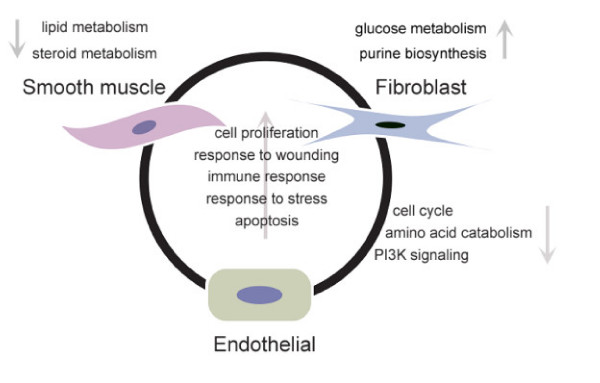
**Summary of common and cell specific responses elicited in human cells infected with *T. cruzi***. Common transcriptional responses are shown inside the circle, while individual responses are shown in the outside, next to each cell type. Transwell data was not available for VSMC. Arrows indicate up or downregulation.

With the goal of expanding the current understanding of intracellular requirements of *T. cruzi *and the host pathways and processes that support its intracellular growth, an experimental approach that combined transcriptional profiling and transwell culture systems was employed in this study. A core set of host transcriptional changes elicited directly (independently from soluble factors, i.e. 'cytokines') by intracellular infection with *T. cruzi *in three different human cell types was unveiled (summarized in Figure 5). Common responses include metabolic and signaling pathways involved in cell proliferation, amino acid catabolism and response to wounding. The transcriptional profiles also suggested the blockage of the cell cycle progression due to parasite infection in two out of the three studied cell types, a novel finding that was experimentally confirmed as infected cells demonstrated a failure to undergo cytokinesis. The experimental approach employed in this study is a valid tool to unveil biological events triggered directly by the presence of intracellular microorganisms, without the mediation of soluble factors.

## Methods

### Cell line and parasite maintenance

LLcMK_2 _cells and human foreskin fibroblasts (HFF) were maintained in DMEM supplemented with 10% fetal bovine serum (FBS), 2 mM glutamine, 100 U/ml penicillin and 100 μg/ml streptomycin. Human vascular smooth muscle cells (VSMC) were grown in Ham's F12K medium with 2 mM L-glutamine, 1.5 g/L sodium bicarbonate, 10 mM HEPES, 10 mM TES, 0.05 mg/ml ascorbic acid, 0.001 mg/ml insulin, 0.01 mg/ml transferrin, 10 ng/ml sodium selenite, 0.03 mg/ml endothelial cell growth supplement (ECGS) and 10% FBS. Human cardiac microvascular endothelial cells (HMVEC) were maintained in endothelial cell basal medium-2 and endothelial cell growth supplements (Cambrex Bio Science) including 5% FBS. Y-strain *T. cruzi *trypomastigotes were maintained by serial passage in LLcMK2 cells as previously described [54].

### Experimental *T. cruzi *infection

To achieve 80% confluence 48 hrs post-plating VSMC were seeded at a density of 2 × 10^5 ^cells/10 cm dish. For Transwell experiments, the lower wells of 10 cm Transwell plates (Corning Inc; 3 μm pore size) were seeded at a density of 2 × 10^5 ^cells/dish (HFF) or 4 × 10^5 ^cells/dish (HMVEC) with half the amount, respectively, plated in the upper chamber. VSMC or HFF/HMVEC in the lower chamber of Transwell were infected for 2 hours with 1 × 10^8^/ml trypomastigotes at 37°C in a CO_2 _incubator in the appropriate growth medium for each cell type supplemented with 2% FBS. Extracellular parasites were aspirated, and infected monolayers rinsed 5 times with PBS before incubation in complete growth medium (10% FBS for VSMC and HFF and 5% FBS for HMVECs) for the indicated times. To monitor infection levels, sterile 12 mm^2 ^coverslips, placed in dishes prior to cell seeding, were removed, fixed in 2% w/v paraformaldehyde/PBS and immunostained as described [30]. Control monolayers were mock-infected in 2% FBS medium and submitted to the same washing steps as the experimental infections.

### RNA extraction

Cell monolayers were rinsed 3 times with sterile PBS and total RNA was isolated with Trizol reagent (Invitrogen) in accordance with the manufacturer's instructions and concentrated using the RNeasy kit (Quiagen). RNA was quantitated spectrophotometrically in a Beckman DU 530 spectrophotometer and stored at -80°C.

### Microarray hybridization and analysis

RNA quality control and microarray hybridization was performed at the at the Harvard Medical School's Biopolymers facility. RNA integrity was evaluated employing an Agilent 2100 Bioanalyzer (Agilent Technologies), and 5 μg of each sample were processed and hybridized onto HG_U133 plus 2.0 Affymetrix chips, according to manufacturer's specifications for One-Cycle Target labeling Assay. Data acquisition performed in a GeneChip^® ^Scanner 3000 (Affymetrix). The Affymetrix data files containing the unprocessed intensity values (i.e. Affymetrix .cel files) were imported into Rosetta Resolver Biosoftware 7.0 where the data was pre-processed to reduce systematic errors (i.e. background subtraction and intra-array normalization). Through the application of the Affymetrix-specific error model within Rosetta Resolver [55], the data were transformed into profiles (i.e. scanned, imaged and normalized expression data). Intensity ratios were calculated from the statistical combination of replicate profiles in order to increase the confidence in measurements, and to obtain fold-change values and associated *ρ-*values. Transcripts showing at least a 2-fold change (*ρ *< 0.01) with respect to their matched controls were used to create gene lists that were subsequently uploaded into Genespring GX gene expression analysis software (Agilent Technologies) to generate heat maps and perform GO function analysis or into Ingenuity Pathway Analysis™ (IPA) (Ingenuity Systems Inc.) software to identify metabolic and signaling pathways. Redundant probes (more than one probe corresponding to the same gene) within the gene sets employed in the GO function analysis were manually removed to avoid inflating the overlap with GO categories. In accordance to the minimum information about microarray experiment (MIAME) guidelines, the complete raw and processed data files for each array are publicly available at the Gene Expression Omnibus (GEO) database repository  [GEO: GSE13791].

### Quantitative RT-PCR

Total RNA was treated with DNAse (Invitrogen) and reverse transcribed using the Iscript kit (Bio Rad). Quantitative PCR was performed in a 7300 real-time PCR system (Applied Biosystems) using real time PCR mastermix (ABI). Specific primers and FAM-labeled probes for each gene of interest were purchased as inventoried assays from Applied Biosystems. ΔΔCT values were calculated with respect to VIC-labeled human GAPDH controls (ABI) and data is represented as fold-change for individual genes under experimental (infection, exposure to soluble factors) relative to mock-infected control cells. The samples employed for the qPCR analysis included the same samples used in experiments analyzed by DNA microarray hybridization as well as additional independent experiments.

### Quantification of cytokine production by infected cells

HFFs, HMVECs and VSMC were seeded in six-well plates and mock/infected as described above. Aliquots of 120 μl of medium were taken every 6 hours, filter sterilized and analyzed using SearchLight custom Multiplex arrays (Pierce Biotechnology Inc.).

### Cell cycle progression analysis

Cells were seeded into two 6-well plates, allowed to grow and reach confluence for 48 hours prior to being mock/infected as described above. FACS analysis of propidium iodide stained cells showed that this strategy induced the arrest of most of the cell population into Go phase of the cell cycle. Six hours post infection the cells were re-split into 6-wells containing sterile cover slips at a 1:4 ratio and allowed to re-attach for 12 hours. To monitor entry into S-phase, cover slips were stained with BrdU using the *in situ *cell proliferation kit FLUOS (Roche) according to manufacturer's instructions. Parasite and host cell DNA was counterstained with propidium iodide prior to mounting the slides with Mowiol. To evaluate progression into M-phase, fixed cells were, stained with anti-human phospho-histone H3 (Ser 10) antibody (Cell Signaling Technology) and host cell and parasite DNA were counterstained with DAPI. Images were obtained with a CCD camera mounted on a Nikon TE-300 epifluorescescence microscope and analyzed employing MetaMorph software (Universal Imaging Corporation).

### Cell fusion experiment

HFFs were seeded into two 6-well plates, allowed to grow for 48 hours and mock/infected as described above. Six hours post-infection, each plate was loaded with 5 μM of either Cytotracker™ green or Cytotracker™orange dye (Invitrogen) for 45 minutes in accordance with manufacturer's instructions. The cells were then trypsinized, green and orange dyed cells were mixed, and plated into 6-well plates containing sterile cover slips at one fourth the density of the original cultures. At 24, 48 and 72 hours, post infection, cover slips were removed, fixed with 3.7% formaldehyde in PBS for 15 minutes, counterstained with DAPI, mounted with MOWIOL and visualized as described above.

## Abbreviations

**Akt**: v-akt murine thymoma viral oncogene homolog 1; **ARPC1A**: actin related protein 2/3 complex, subunit 1A, 41 kDa; **B3GNT2**: UDP-GlcNAc:betaGal beta-1,3-N-acetylglucosaminyltransferase 2; **B4GT5**: UDP-Gal:betaGlcNAc beta 1,4-galactosyltransferase, polypeptide 5; **BCAT2**: branched chain aminotransferase 2, mitochondrial; **BrdU**: bromodeoxyuridine; **CARKL**: carbohydrate kinase-like; **CCL5**: chemokine (C-C motif) ligand 5 also known as RANTES;**cDNA**: complementary deoxyribonucleic acid; **CH25H**: cholesterol-25-hydroxylase; **CoA**: coenzyme A; **CTMP**: thioesterase superfamily member 4; **DAPI**: 4',6-diamidino-2-phenylindole; **DLG1**: discs, large homolog 1; **DMEM**: Dulbecco's modified Eagle's medium; **DNA**: deoxyribonucleic acid; **DNAse**: deoxyribonuclease; **ECGS**: endothelial cell growth supplement; **EGF**: epidermal growth factor (beta-urogastrone); **FAM**: 6-carboxyfluorescein; **FARP1**: FERM, RhoGEF (ARHGEF) and pleckstrin domain protein 1; **FBS**: fetal bovine serum; **FGF**: fibroblast growth factor; **GAPDH**: glyceraldehyde-3-phosphate dehydrogenase; **GO**: gene ontology; **GPI**: glycosyl phosphatidylinositol; **HEPES**: N-2-hydroxyethylpiperazine-N'-2-ethanesulfonic acid; **HFF**: human foreskin fibroblast; **HIP1R**: huntingtin-interacting protein 1-related protein; **HMVEC**: human microvascular endothelial cells; **IFNβ**: interferon beta; **IL-11**: interleukin 11; **IL-15**: interleukin 15; **IL-6**: interleukin 6; **IL-8**: interleukin 8; **ISG20**: IFN-stimulated exonuclease; **ISGs**: interferon stimulated genes; **KLF4**: kruppel-like transcription factor-4; **LLcMK**_2_: rhesus monkey kidney epithelial cells; **MOWIOL**: poly(vinyl alcohol); **mTOR**: mammalian Target of rapamycin; **MX1**: myxovirus resistance protein; **MYH10**: myosin, heavy chain 10; **MYOCD**: myocardin, **OAS1**: 2,5-oligoadenylate synthase; **PANK2**: pantothenate kinase 2; **PBS**: phosphate buffered saline; **PCR**: polymerase chain reaction; **PDGF**: platelet-derived growth factor; **PFK**: phosphofructokinase; **PI3K**: phosphatidylinositol-3-Kinase; **PNP**: purine nucleoside phosphorylase; **PP2A**: protein phosphatase 2A; **qPCR**: quantitative polymerase chain reaction; **RNA**: ribonucleic acid; **RSAD2**: radical S-adenosyl methionine domain-containing protein 2; **SLC39A8**: solute carrier family 39 member 8; **SPHK1**: sphingosine kinase-1; **SPP1**: secreted phosphoprotein 1 (osteopontin, bone sialoprotein I, early T-lymphocyte activation 1); **SSH2**: slingshot homolog 2; **STAT-1**: signal transducer and activator of transcription 1; **STIM1**: stromal interaction molecule 1; **SYTL3**: synaptotagmin-like 3; **TES**: N-Tris(Hydroxymethyl)methyl-2-Amino-Ethanesulfonic Acid; **TMOD 3**: tropomodulin 3; **TSC2**: tuberous sclerosis 2; **T-SNARE1**: t-SNARE domain containing 1; **UHRF1 protein**: ubiquitin-like, containing PHD and RING finger domains 1;**VEGF**: vascular endothelial growth factor; **VSMC**: vascular smooth muscle cells; **WIRE**: WAS/WASL interacting protein family, member 2.

## Authors' contributions

JAC conducted all experimental work, carried out microarray analysis and drafted and manuscript. JPD was involved in microarray and bioinformatic analysis as well as manuscript preparation. BAB conceived the study and design, was involved in pathway analysis and edited manuscript. All authors have read and approved the final manuscript.

## Authors' information

JAC is an Assistant Professor at the Center for Infectious Disease Research, Biological Sciences School, Pontifical Catholic University of Ecuador. Address: Edificio de Química, PB# 004, Av. 12 de Octubre y Carrión, Quito-Ecuador.

JPD is an Associate Professor of Medicine at Albert Einstein College of Medicine, 1301 Morris Park Avenue, Bronx, NY 10461.

BAB is an Associate Professor at Harvard School of Public Health. Address: Harvard School of Public Health, 665 Huntington Avenue, Boston MA 02115.

## Supplementary Material

Additional file 1 **Table S1 – Gene expression changes in *T. cruzi*-infected VSMC 24 hr**. Gene expression changes occurring in human vascular smooth muscle cells 24 hours after infection with *T. cruzi*. The data shown corresponds to probes for transcripts showing an intensity increase or reduction of 2-fold or greater (p < 0.01) with respect to their matched controls according to the analysis with Rosetta Resolver Biosoftware 7.0. Click here for file

Additional file 2 **Table S2 – Gene expression changes in *T. cruzi*-infected HFF 24 hr**. Gene expression changes occurring in human foreskin fibroblasts 24 hours after infection with *T. cruzi*. The data shown corresponds to probes for transcripts showing an intensity increase or reduction of 2-fold or greater (p < 0.01) with respect to their matched controls according to the analysis with Rosetta Resolver Biosoftware 7.0. Click here for file

Additional file 3 **Table S3 – Gene expression changes in *T. cruzi*-infected HMVEC 24 hr**. Gene expression changes occurring in human microvascular endothelia cells 24 hours after infection with *T. cruzi*. The data shown corresponds to probes for transcripts showing an intensity increase or reduction of 2-fold or greater (p < 0.01) with respect to their matched controls according to the analysis with Rosetta Resolver Biosoftware 7.0. Click here for file

Additional file 4 **Table S4 – *T. cruzi*-induced genes common to VSMC, HFF and HMVEC 24 hr**. Gene expression changes at 24 hours after infection with *T. cruzi *that are common to human foreskin fibroblasts, human microvascular endothelial cells and human vascular smooth muscle cells. The data shown corresponds to probes for transcripts that show an intensity increase of 2-fold or greater (p < 0.01) with respect to their matched controls according to the analysis with Rosetta Resolver Biosoftware 7.0. Click here for file

Additional file 5 **Table S5 – GO function categories associated with *T. cruzi*-induced genes**. Gene ontology analysis was performed using Genespring GX to identify functions with significant overlap (p < 0.05, overlap = 5 genes) with the genes upregulated in human foreskin fibroblasts, human microvascular endothelial cells and human vascular smooth muscle cells 24 hours after infection with *T. cruzi*. The functions induced in each cell type were intersected, and common functions are shown. Click here for file

Additional file 6 **Table S6 – Cytokine/chemokine genes upregulated in *T. cruzi *infected cells**. Gene expression changes (2-fold or greater p < 0.01) occurring in each of the studied cell types for cytokine and chemokine genes with respect to their matched controls according to the analysis with Rosetta Resolver Biosoftware 7.0. Click here for file

Additional file 7 **Table S7 – 'Cytokine'-independent genes modulated by *T. cruzi *infection of HFF**. Gene expression changes occurring in human foreskin fibroblasts 24 hours after infection with *T. cruzi*, after removal of genes induced by soluble factors through the use of transwells, as described in methods. The data shown corresponds to probes for transcripts showing an intensity increase or reduction of 2-fold or greater (p < 0.01) with respect to their matched controls according to the analysis with Rosetta Resolver Biosoftware 7.0. Click here for file

Additional file 8 **Table S8 – 'Cytokine'-independent genes modulated by *T. cruzi *infection of HMVEC**. Gene expression changes occurring in human microvascular endothelial cells 24 hours after infection with *T. cruzi*, after removal of genes induced by soluble factors through the use of transwells, as described in methods. The data shown corresponds to probes for transcripts showing an intensity increase or reduction of 2-fold or greater (p < 0.01) with respect to their matched controls according to the analysis with Rosetta Resolver Biosoftware 7.0. Click here for file

Additional file 9 **Table S9 – Common GO function categories for cytokine-independent responses in *T. cruzi*-infected HFF and HMVEC**. Gene ontology analysis was performed using Genespring GX to identify functions with significant overlap (p < 0.05, overlap = 5 genes) with the genes up or downregulated in human foreskin fibroblasts or human microvascular endothelial cells 24 hours after infection with *T. cruzi*. The functions modulated in each cell type were intersected, and common functions are shown. Click here for file

Additional file 10 **Table S10-Genes in the top GO function categories representing upregulated genes in *T. cruzi*-infected HFF and HMVEC**. Gene ontology analysis was performed using Genespring GX to identify functions with significant overlap (p < 0.05, overlap = 5 genes) with the genes up or downregulated in human foreskin fibroblasts or human microvascular endothelial cells 24 hours after infection with *T. cruzi*. The genes in the two top functional categories upregulated genes in HFF and HMVEC are shown. Click here for file

Additional file 11 **Table S11 – Gene expression changes in *T. cruzi-*infected VSMC 48 hr**. Gene expression changes occurring in human vascular smooth muscle cells 48 hours after infection with *T. cruzi*. Shown are genes with an intensity reduction of 2-fold or greater (p < 0.01) with respect to their matched controls according to the analysis with Rosetta Resolver Biosoftware 7.0. Click here for file
